# Silent Hypoxia in COVID-19: A Case Series

**DOI:** 10.1159/000520083

**Published:** 2021-11-26

**Authors:** Thomas Fuehner, Isabelle Renger, Tobias Welte, Tobias Freundt, Jens Gottlieb

**Affiliations:** ^a^Department of Respiratory Medicine, Siloah Hospital, Hannover, Germany; ^b^Dept of Respiratory Medicine and German Centre for Lung Research (DZL/BREATH), Hannover Medical School, Hannover, Germany

**Keywords:** SARS-CoV-2, Coronavirus disease 2019, Dyspnea, Hypoxemia, Hypocapnia

## Abstract

**Background:**

The coronavirus disease 2019 (COVID-19) pandemic is an ongoing global crisis challenging the worldwide healthcare systems. Many patients present with a mismatch of profound hypoxemia and few signs of respiratory distress (i.e., silent hypoxemia). This particular clinical presentation is often cited, but data are limited.

**Main Body:**

We describe dyspnea sensation as assessed by using the BORG scale in pulmonary patients admitted to the emergency room during a 4-week period and transferred to the respiratory department of Siloah Hospital, Hannover, Germany. From October 1 to November 1, 2020, 82 patients with hypoxemia defined as oxygen demand to achieve an oxygen saturation (SpO<sub>2</sub>) ≥92% were included. In 45/82 (55%) patients, SARS-CoV-2 was detected by PCR on admission. Among non-COVID patients, exacerbation of COPD was the main diagnosis (15/37, 41%). All subjects rated their perceived dyspnea using the modified Borg CR10 scale. Patients in the non-COVID group suffered from more dyspnea on the modified Borg CR10 scale (median 1, IQR: 0–2 vs. median 5, IQR: 3–6, *p* < 0.001). In multivariate analysis, “silent hypoxemia” as defined by the dyspnea Borg CR10 scale ≥5 was independently associated with COVID-19 and presence of severe hypocapnia with an odds ratio of 0.221 (95% confidence interval 0.054, 0.907, *p* 0.036).

**Conclusion:**

Among pulmonary patients with acute hypoxemia defined as oxygen demand, patients suffering from COVID-19 experience less dyspnea compared to non-COVID patients. “Silent” hypoxemia was more common in COVID-19 patients.

## Introduction

In COVID-19 patients, hypoxemia with a disproportional low sensation of dyspnea has been described. This phenomenon is referred as “silent” or “happy” hypoxemia [1, 2, 3]. The underlying mechanism of silent hypoxemia is not fully understood. “Silent” hypoxemia is not limited to COVID-19 and can be found in other respiratory disorders. The exact proportion of patients with silent hypoxemia in COVID-19 and other respiratory diseases is unknown. The aim of this study was to investigate the sensation of dyspnea in hypoxemic emergency admissions.

## Method

All emergency admissions to the respiratory care unit in Siloah Hospital, Hannover, Germany, between October 1 and November 1, 2020, were analyzed retrospectively. All patients received SARS-CoV-2 PCR testing on admission. Patients with hypoxemia defined as supplementary oxygen demand to achieve an oxygen saturation (SpO_2_) ≥ 92% in the first 24 h after admission were included. Vital signs were recorded, and the national early warning score (NEWS-2) was used for monitoring [4]. Oxygen demand was titrated by capillary blood gas analysis [5]. Subjects rated their perceived dyspnea using the modified Borg CR10 scale in license [6], Agreement ID 13LX473. This scale has 12 stages from 0 to 10 including 0.5. Numbers on this scale are related to a description of dyspnea during exertion. Demographics, chronic conditions (cancer, diabetes mellitus, hypertension, chronic respiratory disease, and obesity), signs and symptoms, including fever, cough, anosmia, ageusia, dyspnea, and oxygen saturation, and results of blood gas analysis were analyzed. The study was performed in accordance with the ethical guidelines of the 1975 Declaration of Helsinki and the institutional review board.

### Statistics

Continuous variables were described as median and 25 and 75% quartiles. Categorical variables are presented as *n* (%). χ^2^ test or Fisher's exact test for categorical variables if appropriate and Student's test for metric variables after testing for normal distribution to compare groups were used. A logistic regression model was fitted with a Borg CR10 scale of ≥5 as the categorical outcome. Variables included were identified by a *p* value of <0.1 in univariate analysis.

## Results

**Fig. 1:**
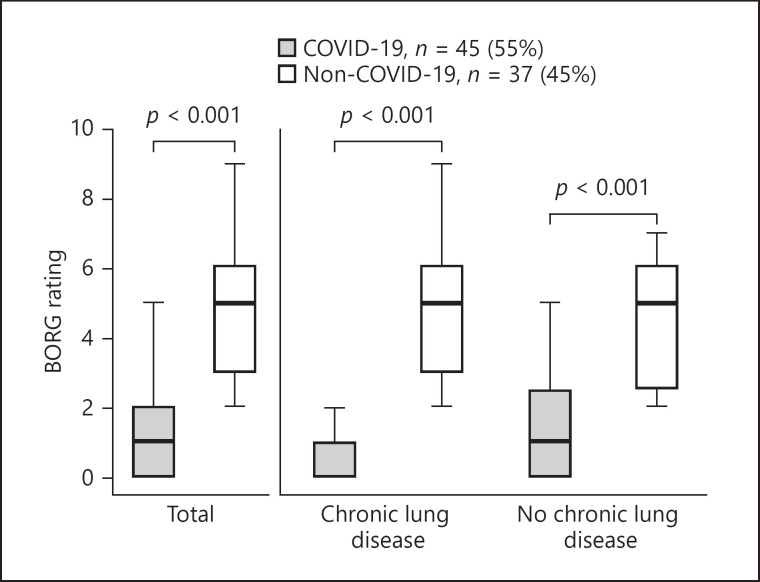
Dyspnea rated by using the Borg CR10 scale in emergency admissions.

During the study period, 82 patients were included. In 45/82 (55%) patients, SARS-CoV-2 was detected by PCR on admission. In 9 out of 45 (20%) of COVID-19 patients, a chronic lung disease was present compared to 28/37 (76%) patients from the non-COVID group (Table 1). Two out of 37 (5%) patients in the non-COVID group were using domiciliary long-term oxygen treatment prior to hospitalization. Among non-COVID patients, the majority (15/37, 41%) suffered from acute exacerbation of COPD, followed by community-acquired pneumonia (8/37, 22%). More patients in the non-COVID had a pCO_2_ ≥45 mm Hg. No COVID-19 patient was hypercapnic. Dyspnea rated by the Borg CR10 scale was lower in the COVID-19 group, including those with chronic lung disease (Fig. 1). All but 1 non-COVID-19 patient (98%) had a Borg CR10 scale of <5. There was no difference in hospital length of stay (median 10 vs. 8 days, *p* = 0,189), ICU admission (7/45 vs. 8/37, *p* = 0.48), invasive ventilation (2/45 vs. 2/37, *p* = 1.0), or mortality (6/45 vs. 2/37, *p* = 0.28) between COVID and non-COVID patients. In multivariate analysis, silent hypoxemia as defined by the dyspnea Borg CR10 scale ≥5 was independently associated with COVID-19 and presence of severe hypocapnia with an odds ratio of 0.221 (95% confidence interval 0.054, 0.907, *p* 0.036) (Table 2).

## Discussion

To our knowledge, this is the first series which systematically analyzes the sensation of dyspnea in COVID-19 patients compared to patients with other respiratory diseases. Patients with COVID-19 had a higher dyspnea sensation as assessed by the BORG scale when the oxygen partial pressure was similar.

Dyspnea as shortness of breath is frequently reported in community-acquired pneumonia and COPD exacerbations in 75% and 78%, respectively [7, 8]. Docherty et al. [9] reported in a large observational cohort of 20,133 hospital admissions with COVID-19 shortness of breath in 71%. In contrast and similar to our results, in 1,712 COVID-19 inpatients (two-thirds with pneumonia), 65% did not complain of a shortness of breath at admission [10]. Unfortunately, these publications did not use an exact definition of breathlessness or did not objectively rate dyspnea.

Hypoxemia in COVID-19 is caused by intrapulmonary shunts, loss of lung perfusion regulation, intravascular microthrombi, impaired diffusion capacity, and preservation of lung mechanics [11, 12, 13, 14]. Interestingly, the respiratory compensation mechanisms differ significantly according to the underlying lung pathology. Patients with COVID-19 and hypoxemia tend to compensate by hyperventilation and are usually hypocapnic as confirmed in our study. This respiratory compensation mechanism seems to be similar to community-acquired pneumonia or interstitial lung diseases. In both entities, dyspnea is a major symptom. Recently, hypocapnic hypoxemia without dyspnea was thought to be caused by right-to-left intrapulmonary shunt in COVID-19 [14, 15].

Hypoxemia has a limited correlation with the sensation of breathlessness while dyspnea is more correlated with hypercapnia [16, 17]. “Silent hypoxemia” in COVID-19 may be caused by changes in the respiratory control system. Angiotensin-converting-enzyme 2 receptors are widely expressed in the nasal mucosa as well as in carotid bodies, where oxygen chemoreceptors for regulation of respiration are located. Another example of neural disturbances in COVID-19 is anosmia reported by one-third of patients with COVID-19 [10, 18]. Silent hypoxemia in COVID-19 deserves further study to elucidate its mechanism.

The series has potential limitation from the unicenter design with a small number of participants and a following lack of subgroup analysis. Virus variants were not screened in the clinical routine to that time. In addition, the Borg CR10 scale is a tool for measuring an individual's dyspnea during physical work. The scale is not established to rate dyspnea in patients with acute hypoxemia at rest.

## Conclusion

Silent hypoxemia is more common in COVID-19 patients compared to pulmonary patients with acute hypoxemia. Further evaluation of its uniqueness and pathophysiologic mechanisms is needed.

## Statement of Ethics

Data from the clinical routine were analyzed retrospectively. The study was performed in accordance with the Institutional Review Board, KRH Klinikum Region Hannover. All procedures, including the informed consent process, were conducted in accordance with the ethical standards of the responsible committee on human experimentation (institutional and national) and with the Helsinki Declaration of 1975, as revised in 2000.

## Conflict of Interest Statement

The authors have no conflicts of interest to declare.

## Funding Sources

No funding was received.

## Author Contributions

T.F. participated in the design of the study, performed the statistical analysis, conceived of the study, participated in its design and coordination, and helped to draft the manuscript. I.R. participated in the design of the study, conceived of the study, participated in its design and coordination, and helped to draft the manuscript. T.W. conceived of the study, participated in its design and coordination, and helped to draft the manuscript. T.F. participated in the design of the study, conceived, and helped to draft the manuscript. J.G. participated in the design of the study, performed the statistical analysis, conceived of the study, participated in its design and coordination, and helped to draft the manuscript. All authors read and approved the final manuscript.

## Data Availability Statement

The software, databases, and application/tool described in the manuscript are available for testing by reviewers.

## Figures and Tables

**Table 1:** Clinical characteristics

	Total	COVID-19	Non-COVID-19	*p* value
*N* (%)	82 (100)	45 (55)	37 (45)	
Gender female, *n* (%)	26 (32)	15 (33)	11 (30)	0.727
Age, median (25, 75 percentile), years	74 (63, 80)	69 (57, 78)	76 (68, 82)	0.032
Chronic respiratory disease, *n* (%)	37 (45)	9 (20)	28 (76)	<0.001
COPD	17 (21)	2 (4)	15 (41)	<0.001
Asthma	6 (7)	3 (7)	3 (8)	1.000
Lung cancer	9 (11)	4 (9)	5 (14)	0.501
ILD	6 (7)	−	6 (16)	0.006
Main diagnosis, *n* (%)				
COVID-19	45 (55)	45 (100)		
Malignancy	6 (7)		6 (16)	
Community-acquired pneumonia	8 (10)		8 (22)	
AECOPD	13 (16)		13 (35)	<0.001
AE-ILD	4 (5)		4 (11)	
AE asthma	2 (2)		2 (5)	
Congestive heart failure	3 (4)		3 (8)	
Oxygen flow rate, median (25, 75 percentile), L/min	2 (2, 4)	2 (2, 4)	2 (2, 4)	0.996
Oxygen saturation, median (25, 75 percentile), %	92 (92, 94)	92 (92, 94)	92 (92, 94)	0.195
Respiratory rate, median (25, 75 percentile)	21 (20, 23)	21 (20, 22)	22 (20, 24)	0.664
Tachypnea, respiratory rate >20/min, *n* (%)	46 (56)	24 (53)	22 (60)	0.578
Temperature, median (25, 75 percentile), °C	37.2 (36.8, 37.8)	37.1 (36.8, 37.7)	37.4 (37, 38)	0.126
Systolic blood pressure, median (25, 75 percentile), mm Hg	130 (120, 140)	130 (120, 140)	130 (120, 145)	0.196
Heart rate, median (25, 75 percentile), bpm	80 (72, 86)	78 (69, 85)	81 (78, 89)	0.014
Early warning score, median (25, 75 percentile)	6 (4, 6)	5 (3, 6)	6 (4,7)	0.020
pH, median (25, 75 percentile)	7.43 (7.40, 7.44)	7.44 (7.40, 7.45)	7.43 (7.35, 7.44)	0.121
BNP, median (25, 75 percentile)	256 (100 1298)	193 (82, 352)	2200 (100, 6,750)	0.022
pO_2_, median (25, 75 percentile), mm Hg	64 (61,66)	64 (61,66)	63 (60, 70)	0.779
pCO_2_, median (25, 75 percentile), mm Hg	33 (31,39)	32 (31,36)	36 (31,50)	0.003
pCO_2_ <35 mm Hg, *n* (%)	48 (59)	32 (71)	16 (43)	<0.001
pCO_2_ ≥45 mm Hg, *n* (%)	15 (18)	−	15 (41)	<0.001
AaDo2, median (25, 75 percentile), mm Hg	109 (98, 140)	107 (100, 161)	110 (95, 136)	0.144
CRP, median (25, 75 percentile), mg/dL	74 (54, 92)	75 (55, 95)	69 (52, 91)	0.699
D-dimer, median (25, 75 percentile)	1.00 (0.86, 2.01)	1.00 (0.89, 1.80)	0.50 (0.50, 7.20)	0.697
Infiltrates	79 (96)	43 (96)	36 (97)	1.000
Specific therapy (multiple items possible), *n* (%)				
Steroids	48 (59)	38 (84)	10 (27)	<0.001
Antibiotic therapy	33 (40)	−	33 (89)	<0.001
Remdesivir	6 (7)	6 (13)	−	0.030

COVID-19, coronavirus disease 2019; COPD, chronic obstructive pulmonary disease; ILD, interstitial lung disease; AE, acute exacerbation, mm Hg, millimeters of mercury; bpm, beats per minute; BNP, brain natriuretic peptide; pO_2_, partial pressure of oxygen; pCO_2_, partial pressure of carbon dioxide.

**Table 2:** Multivariate analysis

BORG ≥5						
covariate	*N*	dyspnea, (n = 22) (28%)	no dyspnea, (n = 56) (72%)	odds ratio	95% confidence interval	*p* value
COVID-19, *n* (%)	78					
No	36 (46)	21 (58)	15 (42)	(Ref)	(Ref)	(Ref)
Yes	42 (54)	1 (2)	41 (98)	0.007	0,000–0.199	0.004
pO_2_, median (25, 75 percentile), mm Hg	78	62 (58, 65)	65 (62, 66)	0.845	0.671–1.063	0.150
pCO_2_, median (25, 75 percentile), mm Hg	78	36 (31, 52)	33 (31, 36)	0.816	0.627–1.061	0.129
Chronic lung disease, *n* (%)	78					
No	41 (53)	5 (12)	36 (88)	(Ref)	(Ref)	(Ref)
Yes	37 (47)	17 (46)	20 (54)	0.705	0.038–13.206	0.815
pH <7.35, *n* (%)	78					
No	68 (87)	15 (22)	53 (78)	(Ref)	(Ref)	(Ref)
Yes	10 (13)	7 (70)	3 (30)	95.816	1.744–5,263-453	0.026
NIV, *n* (%)	78					
No	72 (92)	18 (25)	54 (75)	(Ref)	(Ref)	(Ref)
Yes	6 (8)	4 (67)	2 (33)	4.305	0.280–66.176	0.295
Pneumonia, *n* (%)	78					
No	25 (32)	16 (64)	9 (36)	(Ref)	(Ref)	(Ref)
Yes	53 (68)	6 (11)	47 (89)	1.391	0.060–32.408	0.837
Respiratory rate, median (25, 75 percentile)	78	24 (21, 28)	21 (20, 22)	1.271	0.968–1.669	0.084
Heart rate, median (25, 75 percentile)	78	86 (78, 90)	80 (71, 85)	1.086	0.983–1.199	0.105
Early warning score, median (25, 75 percentile)	78	6 (5, 7)	5 (3, 6)	1.060	0.448–2.507	0.894
AaDo2, median (25, 75 percentile)	78	124 (110, 141)	102 (97, 150)	0.936	0.785–1.116	0.461
O_2_ flow rate, median (25, 75 percentile)	78	3 (2, 4)	2 (2, 4)	6.214	0.052–737.097	0.453

Ref, reference category; COVID-19, coronavirus disease 2019; mm Hg, millimeters of mercury; pO_2_, partial pressure of oxygen; pCO_2_, partial pressure of carbon dioxide.
